# Geographic Distribution of Endemic Fungal Infections among Older Persons, United States^1^

**DOI:** 10.3201/eid1709.101987

**Published:** 2011-09

**Authors:** John W. Baddley, Kevin L. Winthrop, Nivedita M. Patkar, Elizabeth Delzell, Timothy Beukelman, Fenglong Xie, Lang Chen, Jeffrey R. Curtis

**Affiliations:** Author affiliations: University of Alabama at Birmingham, Birmingham, Alabama, USA (J.W. Baddley, N.M. Patkar, E. Delzell, T. Beukelman, F. Xie, L. Chen, J.R. Curtis);; Birmingham VA Medical Center, Birmingham (J.W. Baddley);; Oregon Health and Science University, Portland, Oregon, USA (K.L. Winthrop)

**Keywords:** endemic mycoses, histoplasmosis, coccidioidomycosis, blastomycosis, rheumatoid arthritis, fungi, geographic distribution, United States, dispatch

## Abstract

To investigate the epidemiology and geographic distribution of histoplasmosis, coccidioidomycosis, and blastomycosis in older persons in the United States, we evaluated a random 5% sample of national Medicare data from 1999 through 2008. We calculated national, regional, and state-based incidence rates and determined 90-day postdiagnosis mortality rates. We identified 776 cases (357 histoplasmosis, 345 coccidioidomycosis, 74 blastomycosis). Patient mean age was 75.7 years; 55% were male. Histoplasmosis and blastomycosis incidence was highest in the Midwest (6.1 and 1.0 cases/100,000 person-years, respectively); coccidioidomycosis incidence rate was highest in the West (15.2). On the basis of available data, for 86 (11.1%) cases, there was no patient exposure to a traditional disease-endemic area. Knowledge of areas where endemic mycosis incidence is increased may affect diagnostic or prevention measures for older adults at risk.

## Abstract 2

TOC summary: Incidence was highest for histoplasmosis and blastomycosis in the Midwest and for coccidioidomycosis in the West.

Fungal infections have become an increasing problem for older persons in the United States (*1**–**4*). Compared with years past, older adults today are more likely to be considered for transplantation, receive aggressive regimens of chemotherapy, or take immunosuppressive drugs for rheumatologic or autoimmune diseases. In addition, increasing longevity has enabled older adults to travel and participate in outdoor activities where they might be exposed to opportunistic fungal organisms that they did not encounter in their youth or for which primary immunity has waned. Major opportunistic infections in older adults include the endemic mycoses histoplasmosis, blastomycosis, and coccidioidomycosis. These infections are acquired through inhalation of spores in the environment and are often associated with outdoor activities and geographic exposures (*2**,**5**–**8*). Increasing age and decreasing cell-mediated immunity as a result of transplantation, chemotherapy, or other immunosuppressive medications (e.g., tumor necrosis factor–α inhibitors) are the main predisposing factors (*1**,**2**,**9*).

Few data describing the incidence and geographic distribution of endemic mycoses in older persons in the United States are available. For infections such as histoplasmosis or blastomycosis, much of the information regarding geographic distribution of infection was described decades ago for younger adults (*10**,**11*). The most frequently cited study that describes areas of endemicity for histoplasmosis in the United States was published in 1969 by Edwards et al. (*10*). The study identified histoplasmosis endemicity on the basis of histoplasma skin testing, a diagnostic method of unknown sensitivity and probably poor specificity. The study population was military recruits; no older persons were included. In a recent report, Chu et al. described hospitalizations for endemic mycoses in adults and children by using 2002 Nationwide Inpatient Sample Data (*12*). Hospitalization rates for adults were described per region, but incidence rates, specifically for older patients, were not available. Moreover, outpatient cases were not captured; thus, rates were underestimated. Additional contemporary data regarding endemic mycoses among older persons in the United States are needed and would be helpful for identifying disease patterns and the geographic distribution of infection and for targeting areas for focused disease prevention. We describe the geographic distribution of endemic mycoses.

## Methods

We conducted a retrospective cohort study by using 1999–2008 claims data for a random 5% national sample of Medicare beneficiaries. Inclusion criteria for entry into the cohort were as follows: 1) age >65 years at start of follow-up; 2) having full Medicare coverage (parts A and B, not in a Medicare Advantage plan) for at least 13 consecutive months; 3) living in one of the 50 US states or Washington DC; and 4) not having claims for any endemic mycosis during a 12-month period before the start of follow-up (to avoid misclassifying prevalent cases as incident cases). Cohort follow-up began on the earliest date of meeting all eligibility criteria and ended on the earliest of the date of death, loss of full coverage, or diagnosis of endemic mycosis.

### Clinical Data and Definitions

Patient data included demographics, concurrent medical conditions, and diagnosis of endemic mycosis. Endemic mycoses were identified by using codes from the International Classification of Diseases, 9th Revision (ICD-9) (histoplasmosis 115.x, coccidioidomycosis 114.x, blastomycosis 116.x). An incident case of an endemic mycosis required 1 inpatient claim (primary or secondary hospital discharge diagnosis) or at least 2 outpatient claims at least 7 days apart but within 90 days. Two outpatient claims were used to improve definition specificity (*13*). For a subpopulation of the cohort (beneficiaries with Medicare/Medicaid Part D data from 2006 through 2007), outpatient information about antifungal drugs was available. From these data, we developed a more specific case definition and compared results with our primary definition. The more specific case definition was an ICD-9 code for the mycosis plus receipt of a prescription for fluconazole, itraconazole, or voriconazole within 60 days of the diagnosis.

Concurrent medical conditions were identified by use of primary or secondary discharge diagnoses or outpatient visit ICD-9 codes; they were defined as 1 physician claim within 6 months before the case date. Diagnosis codes used for specific conditions are as follows: chronic obstructive pulmonary disease (COPD), 491, 492, 493.2, 496; chronic kidney disease, 585; diabetes mellitus, 250; solid tumor malignancy, 140–199 (excluding 173); hematologic malignancy, 200–208; and neutropenia, 288.0. Mortality rates were determined for 90 days after diagnosis.

### Statistical Analyses

Descriptive statistics were calculated for demographic and clinical characteristics. National, regional, and state-based incidence rates (no. cases/100,000 person-years) were determined for the endemic mycoses. State-specific crude incidence rates for the endemic mycoses were calculated as the number of cases divided by the number of person-years of observation among eligible participants residing in each state. Nationwide incidence was obtained by computing the number of total cases of each endemic mycosis divided by the number of person-years of observation among eligible participants in our sample. Geographic regions were specified as South, Midwest, Northeast, and West on the basis of 2010 Census definitions. The geographic distribution of endemic mycoses was determined by indicating incidences by state on a US map. The primary geographic distribution analysis included all patients in the cohort identified as having an endemic mycosis. A secondary sensitivity analysis, excluding those patients with a change in primary residence in the claims data during the study period, was also performed to determine whether persons who had recently moved affected incidence rates. Finally, cases that occurred outside of traditional endemic areas were identified.

We defined endemicity on the basis of previously published studies describing geographic distribution (*8**,**10**–**12**,**14**,**15*). Histoplasmosis-endemic states were North Dakota, South Dakota, Nebraska, Kansas, Oklahoma, Texas, Minnesota, Iowa, Missouri, Arkansas, Louisiana, Wisconsin, Illinois, Mississippi, Alabama, Kentucky, Tennessee, Indiana, Michigan, Ohio, West Virginia, Pennsylvania, New York, Georgia, North Carolina, and South Carolina. Blastomycosis-endemic states were histoplasmosis-endemic states plus Vermont. Coccidioidomycosis-endemic states were California, Utah, New Mexico, Arizona, Texas, Nevada, and Colorado. Statistical analyses were performed by using SAS version 9.2 (SAS Institute, Inc., Cary, NC, USA).

## Results

### Patient Characteristics

The 5% random Medicare sample comprised 1,913,247 beneficiaries who were eligible for the analysis (Table 1). Among these patients, 775 cases of endemic mycoses were identified (357 histoplasmosis, 345 coccidioidomycosis, 74 blastomycosis), of which 244 (31.5%) were diagnosed by outpatient visit codes only (Table 1). Patient mean age was 75.7 years; 55% of patients were male. Concurrent medical conditions among case-patients with any of the 3 mycoses mentioned above included COPD (34.8%, 95% confidence interval [CI] 28.4%–39.8%), diabetes mellitus (22%, 95% CI 19.9%–27.0%) solid malignancy (16.5%, 95% CI 11.9%–27.0%), and rheumatoid arthritis (5.2%, 95% CI 0–6.1%.) The frequency of underlying solid malignancy was higher among patients with blastomycosis (27%) than among patients with histoplasmosis (18.8%) or coccidioidomycosis (11.9%). In contrast, COPD was more common among patients with histoplasmosis (39.8%) than among patients with coccidioidomycosis (31%) or blastomycosis (28.4%). Mortality rate at 90 days postdiagnosis was 9.5% and was similar for all endemic mycoses.

**Table 1:** Characteristics of Medicare beneficiaries with mycoses, United States, 1999–2008

Characteristic	No. (%) patients			
	Histoplasmosis, n = 357	Coccidioidomycosis, n = 345	Blastomycosis, n = 74	Total, n = 1,913,247*
Male sex	180 (50.4)	200 (58.1)	42 (56.5)	807,204 (42.2)
White race	342 (95.8)	62 (89.9)	61 (82.4)	1,679,198 (87.8)
Region				
Midwest	169 (47.3)	63 (18.3)	29 (39.2)	494,139 (25.8)
North	24 (6.72)	14 (4.0)	2 (2.7)	375,987 (19.7)
South	145 (40.6)	25 (7.25)	41 (55.4)	733,676 (38.4)
West	19 (5.3)	243 (70.4)	2 (2.7)	309,525 (16.2)
Rural location†	137 (38.4)	52 (15.1)	34 (46.6)	502,973 (26.3)
Concurrent medical conditions				
Chronic obstructive pulmonary disease	142 (39.8)	107 (31.0)	21 (28.4)	102,936 (5.4)
Diabetes mellitus	71 (19.9)	80 (23.2)	20 (27.0)	204,726 (10.7)
Solid malignancy	67 (18.8)	41 (11.9)	20 (27.0)	128,766 (6.7)
Hematologic malignancy‡	12 (3.4)	<11	<11	13,393 (0.7)
Rheumatoid arthritis	19 (5.3)	21 (6.1)	0	21,046 (1.1)
Chronic kidney disease‡	20 (5.6)	25 (7)	<11	13,393 (0.7)
Neutropenia‡	<11	<11	<11	3,826 (0.2)
90-day mortality§	35 (9.2)	32 (9.3)	<11	

*Random national sample of 5% of Medicare beneficiaries with claims during 1999–2008; selected for cohort were those who were age >65 years at start of follow-up, had full Medicare coverage (parts A and B, not in a Medicare Advantage plan) for at least 13 consecutive months; lived in the 50 US states or Washington, DC; and did not have claims for any endemic mycosis during a 12-month period before the start of follow-up. Mean age of those with mycoses was 75.7 years. †Rural residential status defined by linkage of 9-digit ZIP codes to the rural-urban commuting area code for the corresponding census block. ‡Per Centers for Medicare and Medicaid Services data use guidelines, cell sizes <11 cannot be shown. §Those who died within 90 days after mycosis diagnosis.

### Incidence Rates

**Figure 1:**
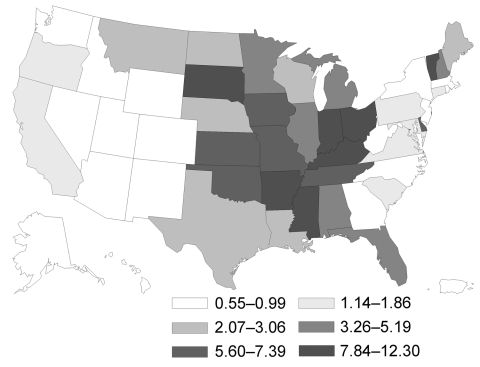
Geographic distribution of histoplasmosis in persons >65 years of age, United States, 1999–2008. Values are no. cases/100,000 person-years.

**Figure 2:**
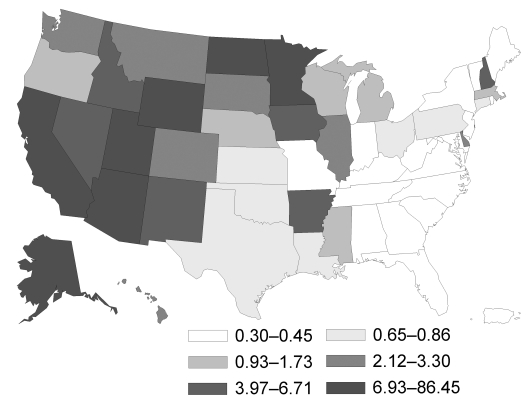
Geographic distribution of coccidioidomycosis in persons >65 years of age, United States, 1999–2008. Values are no. cases/100,000 person-years.

**Figure 3:**
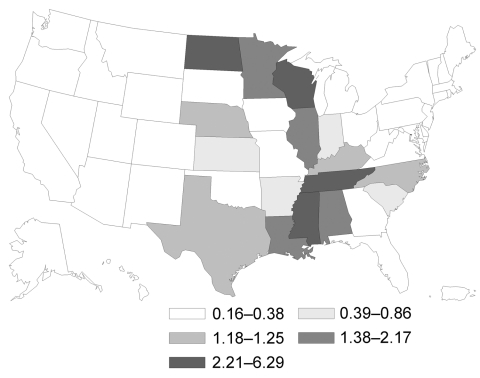
Geographic distribution of blastomycosis in persons >65 years of age, United States, 1999–2008. Values are no. cases/100,000 person-years.

In the United States, the highest incidence rate was for histoplasmosis (3.3) (Figure 1), followed by coccidioidomycosis (3.2) (Figure 2) and blastomycosis (0.7) (Figure 3; Table 2). A geographic distribution was evident (Figure 1). Incidence rates for histoplasmosis were highest for the Midwest (6.1), especially Indiana (13.0) and Arkansas (12.0). Incidence rate for coccidioidomycosis was highest in the West (15.2), especially in Arizona (90.5) and California (10.1). Incidence rate for blastomycosis incidence was greatest in the Midwest (1.0), especially Mississippi (6.4) and Wisconsin (5.7).

**Table 2:** Incidence of endemic mycoses among cohort of Medicare beneficiaries, by region, United States, 1999–2008*

Location	No. cases/100,000 person-years (95% confidence interval)

Histoplasmosis, n = 357	Coccidioidomycosis, n = 345	Blastomycosis, n = 74
Midwest	6.1 (5.3–7.1)	2.0 (1.5–2.6)	1.1 (0.7–1.5)
Northeast	1.1 (0.7–1.7)	0.5 (0.3–0.9)	0.05 (0.01–0.30)
South	3.5 (3.0–4.1)	0.6 (0.4–0.9)	1.0 (0.8–1.4)
West	1.1 (0.7–1.7)	15.2 (13.4–17.2)	0.1 (0.03–0.50)
All of United States	3.4 (3.0–3.7)	3.2 (2.9–3.6)	0.7 (0.6–0.9)

*Random national sample of 5% of Medicare beneficiaries with claims during 1999–2008; selected for cohort were those who were age >65 years at start of follow-up, had full Medicare coverage (parts A and B, not in a Medicare Advantage plan) for at least 13 consecutive months; lived in the 50 US states or Washington, DC; and did not have claims for any endemic mycosis during a 12-month period before the start of follow-up. Mean age of those with mycoses was 75.7 years.

Cases of all 3 mycoses were identified in patients living outside traditional mycosis-endemic areas. To better assess these cases from non–mycosis-endemic areas, we identified whether patients had a medical claim in a traditional mycosis-endemic area during the study period (suggestive of travel to such an area) or previously resided in a mycosis-endemic area (based on residence while enrolled in Medicare). Of 357 histoplasmosis case-patients, 42 (11.8%) had no exposure to a mycosis-endemic area on the basis of available claims data. This finding was similar for blastomycosis (<11/74) and coccidioidomycosis (37/345, 10.7%) cases.

### Alternative Case Definition

During 2006–2007, cases for 17 outpatients were identified by ICD-9 code only; Medicare Part D drug data were available. Among these patients, 10 (59%) had received a prescription for an antifungal drug in the 2 months after diagnosis (2/8 with histoplasmosis, 5/6 with coccidioidomycosis, 3/3 with blastomycosis).

## Discussion

This retrospective cohort study defined geographic distribution of endemic mycoses in older persons in the United States enrolled in Medicare and may help improve diagnostic or prevention measures for those at risk. These endemic mycoses were geographically distributed, but not all occurred in a traditionally mycosis-endemic area. Histoplasmosis was most common, although the highest state-based incidence rates were seen for coccidioidomycosis. As older persons in the United States continue to travel and participate in outdoor activities, exposure to these pathogens may increase. Moreover, increasing age and decreasing cell-mediated immunity as a result of transplantation, chemotherapy, or other immunosuppressive medications increase the risk for endemic mycoses (*1**,**9*). Overall, most cases occurred in patients without known immunocompromising conditions.

Studies estimating US incidence of histoplasmosis, coccidioidomycosis, or blastomycosis are limited, especially among older Americans. Our data suggest that the geographic distribution of these mycoses in older persons in the United States enrolled in Medicare is consistent with prior descriptions for younger patients (*10**,**12*); however, ≈10% of cases were identified in patients with primary residencies outside of mycosis-endemic regions. Our findings of increased incidence of histoplasmosis in the Southeast and Midwest were similar to prevalence estimates with use of skin testing among US Navy recruits (*12*). Our study expands on that early research by identifying cases of histoplasmosis, not antigen sensitivity, in an older population not described previously. Chu et al. reported a similar distribution of infection among children and adults with use of a similar case-finding method but evaluated only hospitalization data, potentially underestimating cases (*12*). Approximately 30% of our cases were identified only by outpatient physician claims.

Other studies have evaluated endemic mycoses in older adults but have not evaluated US geographic distribution (*2**,**4*). Leake et al. reported that coccidioidomycosis was more likely to develop in elderly persons who had recently moved to Arizona (*2*). Blair et al. compared clinical manifestations of coccidioidomycosis among older and younger patients and determined that immunosuppression, independent of age, was a predictor of widespread coccidioidomycosis (*4*). We used a sensitivity analysis and compared the complete cohort and a cohort that did not include patients who moved during the study but found that those who had recently moved did not affect regional incidence rates (data not shown). Of note, ≈10% of patients with an endemic mycosis had not lived or received medical services (based on available claims data) in a traditionally mycosis-endemic area, underscoring the need to consider these infections even in non–mycosis-endemic areas.

In our study population, concurrent conditions were common and, for the most part, similar in frequency among the endemic mycoses. COPD was the most common underlying disease for each endemic mycosis. Chu et al. found that immunosuppression, defined as hematologic or immunologic deficiency or transplantation, was more common in cases of histoplasmosis, when compared with the other endemic mycoses (*12*). Although we did not define immunosuppression as reported by Chu et al., solid malignancy was more frequent in cases of blastomycosis. In most cases, patients were without known immunocompromising conditions. Overall mortality rate for patients with endemic mycoses was low and similar to that seen by Chu et al. (*12*).

The use of Medicare 5% sample data enables national representative estimates of disease occurrence in older Americans, but several limitations deserve mention. The results described from 5% sample Medicare data may not be representative of the entire older American population and may not be valid for other populations outside the United States or for those with other insurance plans. There may be some degree of ascertainment bias because recognition of cases may vary by geographic region. The validity of our identification of presumed cases of endemic mycoses by using ICD-9 codes in claims data are uncertain. Few published data are available that evaluate the positive predictive value of codes for endemic mycoses, compared with other case ascertainment methods, but positive predictive values for opportunistic mycoses approach 70% (*16**–**18*). Our validation, with use of 2006–2007 Medicare Part D drug data for outpatients, suggests that our primary definition is reasonably specific for defining cases of coccidioidomycosis or blastomycosis.

In conclusion, among this cohort of Medicare beneficiaries, histoplasmosis was the most common endemic mycosis. Geographic distribution among older persons in the United States for histoplasmosis, coccidioidomycosis, and blastomycosis is evident, although ≈10% of cases were identified for patients without evidence of claims or residence in traditionally mycosis-endemic areas. Knowledge of areas of increased incidence may improve diagnostic or prevention measures in older adults at risk for endemic mycoses, including those receiving immunosuppressive medications or with new environmental exposures.
